# Toluene and Heavy Metals in Small Automotive Refinishing Shops and Personal Protection of the Workers in Nakhon Si Thammarat, Thailand

**DOI:** 10.1155/2021/8875666

**Published:** 2021-04-16

**Authors:** Udomratana Vattanasit, Jutharat Sukchana, Saowalak Kongsanit, Patjamai Dumtip, Veenuttee Sirimano, Jira Kongpran

**Affiliations:** Department of Environmental Health and Technology, School of Public Health, Walailak University, Nakhon Si Thammarat 80160, Thailand

## Abstract

Chemical contamination and safe work practices of workers in automotive refinishing shops have been extensively studied in industrialized countries, but the evidence in developing countries is limited. This study aimed to evaluate chemical contamination and the use of personal protective equipment (PPE) of workers in local small-scale automotive refinishing shops in Nakhon Si Thammarat, Thailand. Airborne toluene and heavy metals, i.e., lead, chromium, and cadmium, were measured in 3 automotive refinishing shops. Toluene exposure assessed by urinary hippuric acid (*n* = 27) and metal contamination on workers' hands (*n* = 24) were also determined. Information on the use of PPE and personal hygiene practices of the workers was collected by questionnaires. Average ambient levels of toluene (0.04–18.26 ppm) and the metals (Pb: ND-26.34, Cr: 0.02–4.46, and Cd: ND-1.44 *µ*g/m^3^) in all sites did not exceed the national standard levels of 200 ppm for toluene (1998) and 50, 12, and 5 *µ*g/m^3^ for Pb, Cr, and Cd, respectively (2017). The mean ambient levels of these chemicals were highest in paint spray booths followed by nonpainting areas and office rooms, respectively. The highest level of urinary hippuric acid (1.13 g/g creatinine) was found in a painter but did not exceed the recommended biological exposure index of 1.6 g/g creatinine (2014). In contrast, the highest levels of lead and chromium detected on the workers' hands were found in body repair technicians. Direct hand contact without using gloves was suggested as a primary cause of metal contamination.

## 1. Introduction

Automotive refinishing involves the repair and repainting of automobile body parts. Automotive refinishing shops are subjected to national and local regulations mainly due to emissions of toxic substances from paint application processes, which can be hazardous to human health and the environment. Paint is a suspension of pigment particles in a liquid composed of a binder (resin), a volatile solvent or water, and additives that provide the required characteristics of the paint [1]. Among organic solvents used in paints, toluene is a primary solvent that painters are usually exposed to [2–4]. A study in Thailand measured ambient concentrations of benzene, toluene, ethylbenzene, and xylene (BTEX) in 94 automotive paint shops in Bangkok and revealed that toluene was the most abundant BTEX-species [5]. Lead, chromium, and cadmium are toxic metals commonly used in automobile paints as components of paint pigments. Examples of metal pigments include lead oxide (red lead), lead chromate (chrome yellow), chromium oxide (chrome green), and cadmium sulfide (cadmium yellow). Ambient levels of the three metals have been reported in a study in auto body repair shops in Hat Yai City, Songkhla Province, Thailand [6]. The metals can be released during sanding and welding of auto parts and paint spraying. Moreover, scraped car paint dust from the reworking of used vehicles was also proposed as a source of metal exposures [7].

Repeatedly breathing of solvents such as toluene may cause symptoms of neurotoxicity and permanent damage to the brain [8, 9]. Paint pigments can be predominantly absorbed in the lung via inhalation. Dermal absorption of metals is generally low [10]. Ingestion is another exposure route of concern for metals as a result of hand-to-mouth behavior in workers who have poor personal hygienic habits. Inhalation or ingestion of lead causes the same health effects on the brain, kidneys, and reproductive organs. Long-term exposure can cause anemia [11]. Breathing high levels of chromium (VI) can irritate the respiratory tract and cause breathing problems, whereas skin contact can cause skin ulcers and allergic reactions [12]. Inhalation with high levels of cadmium can severely damage the lungs. Long-term ingestion of cadmium at a lower level can lead to an accumulation of cadmium in the kidneys and may cause kidney disease [13]. Additionally, the International Agency for Research on Cancer (IARC) classified the occupational exposures of commercial painting as group 1 carcinogens, based mainly on epidemiological studies that identified increased risks of bladder and lung cancer among painters [14].

Appropriate use of personal protective equipment (PPE) is essential to reduce exposure and possible health effects. The present study aimed to evaluate occupational exposure to toluene and toxic metals, including lead, chromium, and cadmium in local automotive refinishing shops in Nakhon Si Thammarat Province, Southern Thailand. Exposure assessment of toluene by measuring hippuric acid and quantification of the metals in ambient air and hand dust wipe samples were carried out. Information on the use of PPE and personal hygiene practices regarding personal prevention of metal exposure collected by using questionnaires was evaluated. The data obtained could be useful for establishing a national and regional policy to enforce occupational health surveillance programs to protect workers' health.

## 2. Materials and Methods

### 2.1. Study Locations and Subjects

The study sites were automobile body repair shops located in Tha Sala District, Nakhon Si Thammarat Province, Thailand. There were only three out of five registered automobile body repair shops in the district accepted to participate in the study. All the three shops were small enterprises categorized by their service capacity of approximately 30–40 cars/month and the number of workers in each shop (8–14 workers). We divided the automobile body shops into three main areas, including surface body repair area (nonpainting area), painting area, and administrative area (office). The vehicle's structure damage was repaired in the surface body repair area where welding, sanding, and body filler applying took place to make the surface smooth before painting. The shops were operated with a paint spray booth equipped with a ventilation system. All shops open Monday through Saturday from 8 : 30 to 16 : 30. A walk through survey was conducted to get information on workplace characteristics regarding their operations and necessary facilities.

Study subjects were recruited according to their job activities on the day of sampling. The job descriptions were classified as painting tasks (paint mixing and spray painting), nonpainting tasks, and management or office work. There were many duties in the nonpainting tasks; a higher proportion of the subjects in this group were enrolled. All subjects were Thai and healthy. The volunteers were asked in the field for their liver or kidney diseases that could result in abnormal urinary metabolite excretion. The volunteers with the diseases were excluded. The study was divided into two parts. Part I was conducted during August–November 2014. This part involved exposure assessment of toluene by measuring hippuric acid as a biomarker and evaluation of the data obtained from a questionnaire, including personal information (i.e., gender, age, and education), job descriptions, and use of PPE (i.e., clothes, gloves, and face masks). All the workers that accepted to participate in the study were enrolled. A total number of 27 subjects were recruited in this part. Part II was conducted during February–May 2017. It was a determination of lead, chromium, and cadmium in ambient air and hand dust of the workers conducted in the same shops in Part I. A total number of 24 subjects were recruited in this part. They were requested to complete a questionnaire about some personal information (i.e., gender, age, and education), job descriptions, and use of PPE (i.e., clothes, gloves, face masks, and safety shoes), and personal hygiene practices regarding personal prevention of metal exposure. This research was conducted following the Declaration of Helsinki. Part I and Part II of the study were approved by the Human Research Ethics Committee of Walailak University according to protocol number 57/064 and WUEC-16-030–01, respectively.

### 2.2. Part I: Exposure Assessment of Automotive Refinishing Shop Workers to Toluene

#### 2.2.1. Determination of Toluene in Ambient Air

Area air samples were collected throughout the entire eight-hour work shift on a Tuesday at various locations according to job activities, including painting areas (*n* = 3), nonpainting areas (*n* = 3), and administrative offices (*n* = 3). The procedure was conducted according to The National Institute for Occupational Safety and Health (NIOSH) Method 1501 [15]. Briefly, the ambient air sample was collected into a charcoal sorbent tube attached to a personal air sampler set at a flow rate of 200 ml/min for 8 h. In nonpainting areas or offices, a sorbent tube was placed at a height of 1.5 to 2 m in the middle of the room. For painting areas, a sorbent tube was placed at the same height and 1.5 m away from the painting location inside the booth. A field blank was conducted without operating the air pump for quality control in each shop. The charcoal tubes were extracted using 1 ml of carbon disulfide and occasional agitation for 30 min. The extracts were subsequently analyzed for toluene concentration by gas chromatography equipped with a flame ionization detector (GC-FID, Agilent model 6890N, USA). Chromatography was performed on an Rtx-1 column (Crossbond 100% dimethyl polysiloxane, 30 m × 0.32 mm ID *x* 0.25 *µ*m df (Restek, USA). The GC was operated with the following conditions: 99% He as the carrier gas, a 10 : 1 split ratio injection at 220°C, and an oven ramp rate of 25°C/min (the final temperature at 200°C).

#### 2.2.2. Determination of the Urinary Hippuric Acid and Creatinine

Spot urine samples were collected from 27 workers after 2 days of exposure (Tuesday) at the end of the work shift on the same day of the air sampling. The procedure was conducted according to the NIOSH method 8301 [16]. In brief, the urine samples were collected in a polyethylene bottle containing a few crystals of thymol for sample preservation. An aliquot of 1 ml of the well-mixed urine was extracted with 4 ml of ethyl acetate. The organic (upper) layer of 1 ml was evaporated to dryness, and the residue was redissolved with distilled water. The extract was analyzed by high performance liquid chromatography (HPLC, Waters model 2690, USA) equipped with a Photo Diode Array detector (Waters 996, USA) operated at 254 nm. Chromatography was carried out using a LichroCart Lichrospher (100 RP-18 *e*5 *µ*m, Merck, Germany) at 37°C. The mobile phase of water, acetonitrile, and glacial acetic acid (84 : 16 : 0.025% v/v/v) was programmed using an isocratic system with a 1.5 ml/min flow rate at room temperature. Creatinine measurement was conducted for correcting the urinary metabolite levels in concentrated or diluted urine samples. Urinary creatinine levels were determined using the picric acid reaction in alkaline condition (Sigma Diagnostics kit, Sigma-Aldrich, USA). The concentrations of hippuric acid were expressed as *µ*g/l and then converted to g/g creatinine.

### 2.3. Part II: Determination of Lead, Chromium, and Cadmium in Ambient Air and Workers' Hand Dust

#### 2.3.1. Determination of Lead, Chromium, and Cadmium in Ambient Respirable Dust

Respirable dust samples were collected throughout the entire eight-hour work shift on a working day from different areas, including painting areas (*n* = 3), nonpainting areas (*n* = 3), and administrative offices (*n* = 3). Polyvinyl chloride membrane filters (37 mm) with a pore size of 5 *µ*m were equipped in cassette filter holders attached to personal air samplers at a flow rate of 1.5 L/min. A field blank was conducted without operating the air pump for quality control in each shop. The air filter samples were extracted for lead, chromium, and cadmium according to a modified NIOSH method 7300 [17]. Ashing acid (4 : 1 v/v of HNO_3_ and HClO_4_) was used to extract the metals on a hotplate at an internal temperature of 150°C until the solution became clear. The extract was analyzed for the metal concentrations by inductively coupled plasma-optical emission spectrometry (ICP-OES, Perkin Elmer model Avio 200, USA).

#### 2.3.2. Determination of Lead, Chromium, and Cadmium in Hand Dust Wipe

There is currently no description of a reference wiping pattern applicable to the skin. The hand wipe pattern applied in this study was a modification from a method proposed by Gorce and Roff [18]. The hand wiping protocol was explained to all the study subjects. They were asked to do self-wiping on their own hands according to the protocol using a total of two wipes for the left and right hands at the end of the 8 h work shift. Two blank wipes were conducted without wiping for quality control in each shop. The wipe samples were extracted for the metals according to NIOSH method 9102 [19]. Briefly, the wipe samples or blank wipes were extracted using 20 ml of concentrated HNO_3_ and 1 ml of concentrated HClO_4_. The reaction was run for 30 min at room temperature and 2.5 h on a hotplate at an internal temperature of 150°C until the solution became clear. The extract was analyzed for the metal concentrations by ICP-OES (Perkin Elmer model Avio 200, USA).

The correlation coefficient, recovery rate, limit of detection (LOD), and limit of quantification (LOQ) of the analytical procedures are presented in Table 1.

### 2.4. Statistical Analysis

Since a limited number of subjects were available, the sample size of each group of workers was too small to be compared with the other groups by inferential statistics. Descriptive statistics were used to examine the mean and median of numeric data and the frequency of observations as percentages for nominal data. The overall number of the samples included 9 area air samples, 27 postshift urine samples, 9 respirable dust samples, and 24 hand dust wipe samples.

## 3. Results

### 3.1. Subject Characteristics and the Use of PPE

General characteristics of study subjects and information on their use of PPE obtained from the questionnaires in Part I and Part II are presented in Table 2. Most of the subjects were male (85.2 and 83.3%). The average ages of study subjects in Part I and Part II were 36.0 years (range, 22–60 years) and 37.5 years (range, 21–51 years), respectively. The majority of the subjects completed their education at elementary and junior high school levels. The proportion of subjects was highest in nonpainting tasks (59.3 and 70.8% in Part I and Part II, respectively). In Part I, there was a small proportion of the subjects reported the use of long pants and a long-sleeved shirt (18.5%) and chemical-resistant gloves (3.7%). Some of them used cloth face masks or disposable face masks but not chemical cartridge respirators. In Part II, more details on the frequency of using PPE and personal hygiene practices regarding personal prevention of metal exposure were collected. Most of the workers reported the use of long pants and a long-sleeved shirt (83.4%) and face masks (58.3%), but every day use was reported by only 29.2% and 14.3% of them, respectively. However, the masks used were cloth face masks or disposable face masks but not particulate respirators. For other types of PPE, most of the workers never use gloves (66.7%) and safety shoes (54.2%). The types of gloves included fabric and plastic gloves. Only 37.5% of the workers were nonsmokers. Two-thirds of the smokers were daily smokers (41.7%) whereas the rest (20.8%) smoked 1–3 times/week. During the work shift, most of the smokers reported handwashing before smoking (54.5%) but not every day (4–6 times/week). Only 29.2% of the subjects reported that they wash their hands before drinking every day, whereas a higher proportion (75.0%) was found to do that for eating.

### 3.2. Workplace Characteristics

The buildings were opened at least two sides for natural ventilation and no partition wall inside the nonpainting areas. Office rooms for administrative staff and customer services, as well as residential areas of the shop owners, were located in an isolated area in the same building. The office rooms were closed and ventilated with an air conditioning system. A paint spray booth was available, and a mechanical ventilation system was installed in all shops. Field observation data showed that some workers spray paint outside the spray room, especially when they spray small auto body parts. The paint, finishing materials, and solvents were stored in storage cabinets/shelves, but most of them were not kept in proper order.

#### 3.2.1. Part I: Exposure Assessment of Automotive Refinishing Shop Workers to Toluene

As shown in Table 3, the mean ambient level of toluene in painting rooms (14.76 ppm) was 1.57-fold and 113.54-fold higher than the levels in nonpainting areas (9.41 ppm) and office rooms (0.13 ppm), respectively. Besides, the painters had a higher exposure to toluene indicated by an increased mean level of urinary hippuric acid (0.58 g/g creatinine) approximately 2.15-fold higher than nonpainting workers and administrative staffs (0.27 g/g creatinine). The levels of hippuric acid in the urine of painters and nonpainting workers were in accordance with the levels of toluene detected in ambient air. On the other hand, the mean level of hippuric acid in the urine of administrative staff was not in the same trend.

The mean levels of urinary hippuric acid classified by the use of PPE are presented in Table 4. The results showed that urinary hippuric levels of workers who wore gloves or long pants and a long-sleeved shirt and those who did not wear them were at approximately the same level.

#### 3.2.2. Part II: Determination of Lead, Chromium, and Cadmium in Ambient Air and Workers' Hand Dust

The mean levels of ambient lead, chromium, and cadmium were highest in paint spray booths (8.78, 1.76, and 0.48 *µ*g/m^3^) followed by nonpainting areas (4.15, 1.37, and 0.20 *µ*g/m^3^) and office rooms (2.15, 0.98, and 0.07 *µ*g/m^3^), respectively (Table 5). For hand dust wipes, as shown in Table 5, the levels of the metals in hand dust wipes were not in the same trend as found in the ambient respirable dust. The mean levels of lead, chromium, and cadmium contaminated in the hands of workers in nonpainting areas (17.46, 14.17, and 0.36 mg) were 2.96-fold, 14.31-fold, and 1.38-fold higher than those in painters (5.89, 0.99, and 0.26 mg) and 2.51-fold, 34.56-fold, and 0.64-fold higher than those in administrative staffs (6.96, 0.41, and 0.56 mg), respectively.

## 4. Discussion

There are a limited number of researches in Thailand published on the exposure assessment of VOCs in automobile repair shops. This study found that the mean level of airborne toluene was 8.10 ± 6.78 ppm (range, 0.04–18.26 ppm). The highest levels of toluene in all shops were detected in painting areas. Nevertheless, all the ambient levels of toluene did not exceed the recommended standard of 200 ppm TWA according to the notification of the Ministry of Interior, B.E. 2541 (A.D.1998) [20], and the occupational standard of 20 ppm TLV-TWA_8_ [21]. We found that the mean levels of toluene in paint spray booths and nonpainting areas were in accordance with the levels of hippuric acid in the urine of painters and nonpainting workers, respectively. Compared to nonpainting workers, the 1.57-fold higher ambient toluene level in painting rooms (14.76 ± 3.63 ppm) was reflected by the 1.93-fold higher levels of urinary hippuric acid in the painters (0.58 ± 0.38 g/g creatinine). The highest level of the biomarker was found in a painter (1.13 g/g creatinine) who worked in a painting room that contained the highest level of toluene at 18.26 ppm (91.3% of the TLV-TWA_8_). The painters were mainly exposed to paint components during paint mixing and spraying. They were also exposed to the paints that remained in the air and on the paint spray booth surface before and after spraying. According to the field observation data, spraying paint of small auto body parts outside the spray booths as well as disorderly storage of paint cans and solvent containers were likely causes of toluene contamination in the ambient air of nonpainting areas.

The mean level of toluene in office rooms (0.13 ± 0.15 ppm) was 72.38-fold lower than the mean level in nonpainting areas (9.41 ± 2.53 ppm). Interestingly, we found that the mean levels of hippuric acid of nonpainting workers (0.27 ± 0.26 g/g creatinine) and administrative staffs (0.27 ± 0.20 g/g creatinine) were at about the same level. Even though the offices were located in separate areas and the ambient toluene levels were relatively low, the administrative staffs had to walk through the nonpainting areas for cooperative duties. Therefore, they might also be exposed to the same levels of toluene as found in nonpainting workers. However, none of the measured urinary hippuric acid levels exceeded the biological exposure index (BEI) value of 1.6 g/g creatinine [22]. It should be noted that preshift urine samples were not determined in the present study since there was a limitation to collect the samples in the morning. Accordingly, a baseline level of hippuric acid in individuals was not available. The difference in the levels of hippuric acid between preshift and postshift could represent daily exposure more clearly. Overall, the levels of urinary hippuric acid found in the present study (0.02–1.13 g/g creatinine) were consistent with a study by Poongpho (2006) conducted in automobile repair shops in Nakhon Ratchasima Municipality, Thailand (0.03–1.36 g/g creatinine) [23].

Inhalation is considered as the primary pathway for exposure to toluene; however, none of the workers reported the use of chemical cartridge respirators. Moreover, the mean levels of urinary hippuric acid classified by the use of chemical-resistant gloves or long pants and a long-sleeved shirt might imply that wearing the two types of PPE could not reduce exposure to toluene. The gloves were nitrile chemical-resistant gloves with an unsupported cotton liner. In general, they have good resistance to organic solvents. No data on integrity and leakage of the gloves was recorded. The long pants and a long-sleeved shirt that the workers used were an alternative available to prevent skin exposure. However, chemical protective suits with hood should be provided to all painters for better protection.

The level of hippuric acid that was not different following the use of different personal protective devices might be a result of inappropriate selection of the biomarker. Urinary hippuric acid was traditionally applied as a biomarker of toluene exposure in industrial workers. However, the reliability of hippuric acid as a biomarker is limited at low levels of exposure to toluene [24]. Its use may still be valid when exposure to toluene is very high but this is not the case in the present study. Besides, the specificity of hippuric acid is also limited since levels can be modified by benzoic acid, i.e., a major intermediate metabolite of toluene, or its precursors [25]. Consumption of sodium benzoate in processed foods or dietary polyphenols in some fruits and vegetables could result in background urinary hippuric acid in the general population with no occupational exposure to toluene [26, 27]. Based on the concentration likely to be exposed in individuals by inhalation at or below the TLV-TWA of 20 ppm, unchanged toluene and *o*-cresol in the urine are recommended biomarkers of toluene exposure by the American Conference of Governmental Industrial Hygienists [28]. The relationship between data on the use of PPE and levels of the recommended biomarkers should be further investigated.

The levels of lead, chromium, and cadmium in the ambient respirable dust from automobile repair processes found in the present study were less than the recommended standard of 50, 12 (as lead chromate), and 5 *µ*g/m^3^ TWA, respectively, according to the notification of the Department of Labor Protection and Welfare, Ministry of Labor, B.E. 2560 (A.D. 2017) [29]. We noticed that the levels of toluene and metals in ambient air were highest in paint spray booths followed by nonpainting areas and office rooms, respectively. These results suggested that paint is a potential source of ambient toluene and metals in these shops. The ventilation system equipped in paint spray booths and natural ventilation in nonpainting areas were sufficient to maintain the levels of air contaminants below the acceptable limits.

The levels of the metals in hand dust wipes were not consistent with the levels found in the corresponding ambient respirable dust. We observed higher levels of lead and chromium in hand dust wipes of nonpainting workers than those of painters. The nonpainting tasks involved many surface body repair processes which were mostly operated manually by hands. Direct hand-contact with the metal surface of the vehicles and sanding dust during surface preparation without using gloves was a likely cause of the relatively high metal levels in nonpainting workers' hand dust. The metals detected in hand dust wipes reflected an accumulation of metals on workers' hands at the time of sampling (the end of the work shift). The levels might not include all metals that the workers had contacted during the whole work shift. Dermal absorption of metals is generally low [10]. In this context, the major exposure route of concern was ingestion via contaminated hands. The results presented the levels of metals that the workers would be exposed to if they did not wash their hand properly after work. Consistent use of PPE is essential to protect the workers' health. Self-report data on PPE use in this study shows that the majority of workers (66.7%) never use gloves, and no one used gloves every day. The metals detected on workers' hands supported this data. Also, fabric gloves used in these shops could allow some chemicals to pass through onto the skin.

Furthermore, we noticed that some of the workers wore a fabric or disposable dust mask that could filter only coarse particles but not fine aerosols and solvent vapors. Interestingly, most of the workers wore long pants and a long-sleeved shirt (83.4%). However, they wore their own clothes since work uniforms were not provided. Unintentional exposure of their family members to painting materials and other chemical contaminants that might have settled on painters' clothes is possible. Besides the use of PPE, the personal hygiene of the workers is also important to prevent work-related exposure. In this study, most of the smokers (54.5%) report handwashing before smoking. The rest never wash their hands before smoking. Only 29.2% of the workers reported that they wash their hands before drinking every day, whereas a higher proportion (75.0%) was found to do that for eating. As a result, they might be exposed to chemicals contaminated on their hands via ingestion. It has been reported that consumption of vegetables and fruits, for example, tomatoes, berries, onions, garlic, and grapes, which are rich in essential elements (zinc, calcium, and iron) and vitamins (C, B_1_, and B_6_), could prevent lead and cadmium from being absorbed in the body [30]. Therefore, a sufficient intake of the foods should be recommended in the workers that are at risk of lead and cadmium toxicity.

The Public Health Act, B.E. 2535 (A.D.1992) [31], specified that automotive refinishing shops must provide an isolated room for spraying paint. However, standard spray paint booths are not always available in many local small-scale shops or may not consistently be applied by the workers properly. All three small shops enrolled in the present study installed a paint spray booth equipped with a ventilation system. We found that the levels of toluene and metals determined were below the acceptable levels. To minimize the exposure as much as possible, ventilation in the panting booth should continue even after the painting is completed to vent all remaining vapor before workers re-enter the booths which is suggested as a good routine practice for painters. Furthermore, on the job training for painters on proper spraying techniques for better spray transfer efficiency could reduce exposure to paint overspray.

Local automotive refinishing shops in Thailand are mostly small enterprises; accordingly, financial constraints may be a critical factor for small automotive refinishing shops to hire specialists in implementing and monitoring a PPE program. Periodic inspection and strict enforcement of local authorities are suggested as a considerable concern. The workers lack basic education and formal training on workplace hazards and safe work practices. To minimize the health risk of workers and possible health effects, the shop owners should develop and implement an initial training program for all new workers to make them concern about the hazards of chemicals and to encourage appropriate use of PPE and good personal hygiene.

## 5. Conclusions

This study highlights the issue of work-related toxic air pollutants and personal protection of workers in local small-scale automotive refinishing shops in Thailand. The levels of all the air pollutants determined did not exceed the national standard levels established by the Ministry of Interior and the Ministry of Labor. The mean ambient levels of these chemicals were highest in paint spray booths and the highest level of mean toluene exposure represented by urinary hippuric acid was found in painters. On the other hand, the mean levels of the metals detected on the workers' hands were highest in body repair technicians (nonpainting tasks). Direct hand contact with the metal surface of the vehicles and sanding dust during surface preparation without using gloves were proposed as the leading cause. PPE use was not fully implemented by the workers. Training should be conducted to create hazard awareness among workers and minimize exposure of the workers and also their family members from take-home exposure pathways.

## Figures and Tables

**Table 1:** Correlation coefficient, recovery rate, LOD, and LOQ of the analytical procedures.

Sample	Compound	Correlation coefficient	Recovery (%)	LOD (ppb)	LOQ (ppb)
Area air samples	Toluene	0.9980	N/A	2.8	9.3

Urine	Hippuric acid	0.9992	N/A	10.8	36.1

Respirable dust	Lead	0.9999	54.1	4.5	15.0
	Chromium	0.9995	87.6	1.8	6.0
	Cadmium	0.9989	61.6	0.3	1.0

Hand dust wipe	Lead	0.9999	74.0	5.4	18.0
	Chromium	0.9999	83.2	0.9	3.0
	Cadmium	0.9990	80.0	0.6	2.0

**Table 2:** General characteristics of study subjects and information on their use of PPE.

Characteristics	Number of subjects (%)	
	Part I (*n* = 27)	Part II (*n* = 24)
Gender		
Male	23 (85.2)	20 (83.3)
Female	4 (14.8)	4 (16.7)
Age		
20–29	8 (29.6)	5 (20.8)
30–39	12 (44.4)	8 (33.3)
40–49	5 (18.5)	10 (41.7)
50–59	1 (3.7)	1 (4.2)
60–69	1 (3.7)	0 (0.0)
Level of education		
None	2 (7.4)	0 (0.0)
Elementary school	8 (29.6)	9 (37.5)
Junior high school	7 (25.9)	11 (45.8)
Senior high school	4 (14.8)	2 (8.3)
College	6 (22.2)	2 (8.3)
Job description		
Painting tasks	6 (22.2)	3 (12.5)
Nonpainting tasks	16 (59.3)	17 (70.8)
Office work	5 (18.5)	4 (16.7)
Use of PPE		
Long pants and a long-sleeved shirt	5 (18.5)	20 (83.4)
Gloves	1 (3.7)^a^	8 (33.3)
Face masks	0 (0.0)^b^	0 (0.0)^c^
Safety shoes (low shoes)	N/A	11 (45.8)

^a^Chemical-resistant gloves. ^b^Chemical cartridge respirators. ^c^Particulate respirators.

**Table 3:** Ambient levels of toluene and biomarker of toluene exposure in workers.

Study groups	Study parameters					
	Ambient levels of toluene (ppm)^a^			Urinary hippuric acid (g/g creatinine)^b^		
	*n*	Mean ± SD	Median	*n*	Mean ± SD	Median
			(min-max)			(min-max)
Painting tasks	3	14.76 ± 3.63	15.01	6	0.58 ± 0.38	0.54
			(11.01–18.26)			(0.17–1.13)

Nonpainting tasks	3	9.41 ± 2.53	9.01	16	0.27 ± 0.26	0.28
			(7.1–12.12)			(0.02–1.09)

Administrative tasks	3	0.13 ± 0.15	0.05	5	0.27 ± 0.20	0.27
			(0.04–0.30)			(0.04–0.48)

^a^8-hour-average. ^b^Postshift.

**Table 4:** Levels of urinary hippuric acid classified according to the use of PPE.

PPE	Use	Number of workers	Urinary hippuric acid (g/g creatinine)^a^
		(%)	(Mean ± SD)
Chemical-resistant gloves	Yes	1 (3.7)	0.31
	No	26 (96.3)	0.30 ± 0.30

Long pants and a long-sleeved shirt	Yes	5 (18.5)	0.27 ± 0.15
	No	20 (83.4)	0.35 ± 0.33

^a^Postshift.

**Table 5:** Levels of lead, chromium, and cadmium in ambient air and hand dust wipe samples.

Study groups	Metals	Study parameters					
		Ambient levels of metal (*µ*g/m^3^)^a^			Metals in hand dust wipe (mg)^b^		
		*n*	Mean ± SD	Median	*n*	Mean ± SD	Median
				(min-max)			(min-max)
Painting tasks	Pb	3	8.78 ± 15.21	ND	3	5.89 ± 6.22	3.24
				(ND–26.34)			(1.43–13.0)
	Cr	3	1.76 ± 2.35	0.64	3	0.99 ± 0.54	1.09
				(0.17–4.46)			(0.41–1.48)
	Cd	3	0.48 ± 0.83	ND	3	0.26 ± 0.43	0.03
				(ND–1.44)			(ND–0.76)

Nonpainting tasks	Pb	3	4.15 ± 2.40	3.16	17	17.46 ± 19.41	12.96
				(2.40–6.89)			(1.41–66.38)
	Cr	3	1.37 ± 2.10	0.20	17	14.17 ± 4.33	3.43
				(0.12–3.79)			(0.26–16.56)
	Cd	3	0.20 ± 0.16	0.14	17	0.36 ± 0.36	0.19
				(0.09–0.38)			(0.01–1.19)

Administrative tasks	Pb	3	2.15 ± 3.55	0.20	4	6.96 ± 10.71	2.40
				(ND–6.24)			(0.22–22.84)
	Cr	3	0.98 ± 1.37	0.37	4	0.41 ± 0.31	0.32
				(0.02–2.55)			(0.14–0.85)
	Cd	3	0.07 ± 0.08	0.05	4	0.56 ± 0.90	0.16
				(ND–0.16)			(ND–1.90)

^a^8-hour-average. ^b^Postshift.

## Data Availability

The data used to support the findings of this study are included in the article. Raw data are available from the corresponding author upon request.
